# Pilocarpine for Deafness

**Published:** 1885-11

**Authors:** 


### Pilocarpine for Deafness.

For all recent cases of deafness due to labyrinthine disturbances, whatever the primary cause may have been, Politzer tries the subcutaneous injection of a two per cent. solution of muriate of pilocarpine. He injects four drops at first, and gradually increases the dose to ten drops daily. He gets fairly good results in about one-half of the cases. I have seen three cases of persons totally deaf, who, after being treated in this way, could hear and understand loud speeches spoken at the distance of a few inches from the ear ; and Politzer has had one case of perfect recovery of the hearing after it had been absent for three years, and several other very satisfactory results following the use of this drug. He is about to publish the results of his experiments with the history of some of the cases. It is not known how pilocarpine acts in these cases, but the benefit derived from its use is certainly great in some of them.—*Boston Med. and Surg. Journal.*

